# Rutaecarpine Inhibits U87 Glioblastoma Cell Migration by Activating the Aryl Hydrocarbon Receptor Signaling Pathway

**DOI:** 10.3389/fnmol.2021.765712

**Published:** 2021-12-09

**Authors:** Yiyun Liu, Yangsheng Chen, Ruihong Zhu, Li Xu, Heidi Qunhui Xie, Bin Zhao

**Affiliations:** ^1^State Key Laboratory of Environmental Chemistry and Ecotoxicology, Research Center for Eco-Environmental Sciences, Chinese Academy of Sciences, Beijing, China; ^2^University of Chinese Academy of Sciences, Beijing, China

**Keywords:** glioblastoma, rutaecarpine, aryl hydrocarbon receptor, interleukin 24, migration

## Abstract

Glioblastoma is the most frequent and aggressive primary astrocytoma in adults. The high migration ability of the tumor cells is an important reason for the high recurrence rate and poor prognosis of glioblastoma. Recently, emerging evidence has shown that the migration ability of glioblastoma cells was inhibited upon the activation of aryl hydrocarbon receptor (AhR), suggesting potential anti-tumor effects of AhR agonists. Rutaecarpine is a natural compound with potential tumor therapeutic effects which can possibly bind to AhR. However, its effect on the migration of glioblastoma is unclear. Therefore, we aim to explore the effects of rutaecarpine on the migration of human glioblastoma cells U87 and the involvement of the AhR signaling pathway. The results showed that: (i) compared with other structural related alkaloids, like evodiamine and dehydroevodiamine, rutaecarpine was a more potent AhR activator, and has a stronger inhibitory effect on the glioblastoma cell migration; (ii) rutaecarpine decreased the migration ability of U87 cells in an AhR-dependent manner; (iii) AhR mediated the expression of a tumor suppressor interleukin 24 (IL24) induced by rutaecarpine, and AhR-IL24 axis was involved in the anti-migratory effects of rutaecarpine on the glioblastoma. Besides IL24, other candidates AhR downstream genes both associated with cancer and migration were proposed to participate in the migration regulation of rutaecarpine by RNA-Seq and bioinformatic analysis. These data indicate that rutaecarpine is a naturally-derived AhR agonist that could inhibit the migration of U87 human glioblastoma cells mostly *via* the AhR-IL24 axis.

## Introduction

Brain tumors and central nervous system tumors are the third most common cause of cancer death in all histological studies of cancer deaths. According to WHO classification, glioblastoma is the most frequent and aggressive (grade IV) primary astrocytoma in adults (David et al., 2007; Soufiane et al., 2010), with the characteristics of high mortality, low cure rate, and poor prognosis. At present, the standard treatment methods are postoperative radiotherapy and chemotherapy, such as temozolomide (TMZ) therapy (Shabierjiang et al., 2018). However, due to the drug resistance and invasive growth of glioblastoma, the tumor cells gradually lose sensitivity to the chemotherapy and migrate from the primary site to distant brain tissue (Nirmala et al., 2003). In this way, the drug is also difficult to completely remove the mutated tissue after the surgery (Lefranc, 2005). Based on the existing therapeutic technology and method, glioblastoma cannot be completely cured. The median survival time of glioblastoma patients treated by surgery, chemotherapy, or radiotherapy is only about 1 year (Bhageeradh et al., 2015), and the 5-year survival rate is only 4–5% (Mclendon and Halperin, 2003). Developing effective drugs that can inhibit the migration and spread of tumor cells become one of the key strategies for drug development against glioblastoma.

Targeting cell signaling pathways and transduction molecules is an important approach to developing anti-tumor drugs. Many signaling pathways, such as Notch, Hedgehog, Wnt, MAPK et al. have been confirmed to be related to the occurrence and development of cancers (Jing and Bruce, 2005; Naoko et al., 2015; Joseph et al., 2020). Among them, the aryl hydrocarbon receptor (AhR) signaling pathway is closely related to tumor cell migration (Narasimhan et al., 2018). AhR also known as dioxin receptor, is a ligand-activated transcription factor that exists in various species and tissues. Although the abundance of AhR is tissue-specific, the AhR gene sequence is highly conserved in evolution, and the AhR signaling pathway regulates important biological processes (Patricia et al., 2006). Upon the activation *via* binding to certain agonists, the AhR translocates from the cytosol to the nucleus, forms a heterodimer with aryl hydrocarbon receptor nuclear transporter (ARNT), and binds with the corresponding dioxin response element (DRE), which subsequently regulates the transcriptional expression of the downstream genes (Casado et al., 2010). It has been found that the AhR signaling pathway is able to regulate the growth, migration, and invasion of tumor cells (Alexander et al., 2002; Tsai et al., 2017; Jin et al., 2020), and the activation state of AhR plays an important role in the immunotherapy of certain kinds of tumors (Liu et al., 2018; Rahul and Tracy, 2018). *In vivo* and *in vitro* data have shown that the activation of the AhR signaling pathway can inhibit the growth and/or migration of breast cancer (Tao et al., 2011), pancreatic cancer (Jin et al., 2018), and ovarian cancer (Wang et al., 2013). In addition, AhR has become a target for the basic research and drug development of thyroid carcinoma (Gianluca et al., 2015). Besides the aforementioned tumors, the expression of AhR and the activity of the AhR signaling pathway are also related to the tumor characteristics of glioblastoma. Gramatzki et al. (2009) found that the expression of AhR in patient-derived glioblastoma tissues was significantly higher than that in paracancerous tissue. Anthony et al. (2018) found a positive correlation between AhR nuclear localization and histological grade in nine different glioma and meningioma cell lines. High malignant grade tumors have higher AhR expression in nuclear, and the addition of AhR antagonist can significantly reduce the viability of tumor cells. Gramatzki et al. (2009) and Silginer et al. (2016) also proved that AhR can regulate the tumor cell cycle, angiogenesis and influence glioblastoma progression *via* TGF- β pathway. These suggest that constitutive AhR promotes the development of glioblastoma. Recently, it has been found that the activation of the AhR signaling pathway by 2,3,7,8-tetrachlorodibenzo-p-dioxin (TCDD) or 6-formylindolo[3,2-b]carbazole (FICZ) can inhibit the migration of glioblastoma by inducing the expression of downstream target gene Interleukin 24 (IL24) (Liu et al., 2021). IL24 is a tumor suppressor, which can inhibit the migration and invasion of neuroblastoma, lung cancer cells (Zhuo et al., 2013; Janani et al., 2015). Thus, the AhR-IL24 axis was proposed as an important mechanism in the inhibitory regulation of glioblastoma cell migration (Liu et al., 2021). This is consistent with the results that AhR can inhibit tumors after being activated in other kinds of tumors (Li Y. et al., 2014; Yamaguchi and Hankinson, 2019). It seems that the expression of constitutive AhR and the activation of the AhR signaling pathway play different roles in glioblastoma. Thus, finding compounds that can effectively activate the AhR signaling pathway may be a strategy for finding new therapeutic drugs against glioblastoma migration. AhR has a variety of ligands (Drew et al., 2018), among which, TCDD is currently recognized as the most high-affinity ligand of AhR (Maryam and Joann, 2015). The daily diet is one of the sources of human exposure to AhR ligands, and various natural dietary chemicals can directly activate or inhibit the AhR signaling pathway (Yang et al., 2019). Thus, natural compounds with AhR activity may be a good library for screening tumor therapeutic drugs.

Rutaecarpine is an indolopyridoquinazolinone alkaloid isolated from *Evodia rutaecarpa* (Juss.) Benth and related herbs are used for headache, abdominal pain, postpartum hemorrhage, dysentery treatment in clinical (Tian et al., 2019). Pharmacological studies have found that it has high permeability in the blood-brain barrier model of MDCK-pHaMDR cell monolayer, with a variety of neuroprotective effects. Rutaecarpine can improve the Alzheimer’s disease-like pathology and cognitive impairment induced by high glucose in animals, as well as improve learning and memory ability and neurological symptoms (Zhang Y. N. et al., 2018). Rutaecarpine was also reported to reduce the infarct volume and brain water content of mice with cerebral ischemia-reperfusion injury. Therefore, the development and utilization of rutaecarpine as a drug for brain diseases have attracted more and more attention (Lee et al., 2008; Yan et al., 2013; Zhao B. et al., 2020). Gillner et al. (1989) proposed that rutaecarpine has the common molecular structure characteristics of most high-affinity ligands of AhR *via* molecular mapping analysis, after that the AhR-binding ability of rutaecarpine was further evidenced by evaluating the DRE-related transcription activity *via* a reporter gene system and the expression of the AhR downstream gene CYP1A1 in rutaecarpine-treated mouse Hepa-1c1c7 cells. In addition, compared with the other two major compounds derived from *Evodia rutaecarpa*, evodiamine or dehydroevodiamine, rutaecarpine might be a more effective ligand of AhR (Han et al., 2009; Zhang Y. et al., 2018). Therefore, rutaecarpine might be a promising AhR agonist for further evaluating its effect on glioblastoma cell migration. Although rutaecarpine has anti-tumor activity in breast cancer and liver cancer cells (Guo et al., 2016), there are still few reports on its tumor treatment effects on glioblastoma. Based on the species and tissue specificity of AhR ligands in their effects (Denison and Faber, 2017), it is necessary to explore the effect of rutaecarpine on the migration of glioblastoma and its potential molecular mechanism, particularly that related to the AhR pathway. Therefore, in this study, the effects of rutaecarpine on the migration of U87 human glioblastoma cells were explored. The role of AhR in rutaecarpine effects was elucidated, particularly focusing on the newly recognized AhR-IL24 axis.

## Materials and Methods

### Reagents and Drug Treatment

Different stocks of evodiamine (10^–1^–10^–5^ M, Solarbio, Beijing, China), dehydroevodiamine (5 × 10^–2^–10^–5^ M, Solarbio, Beijing, China), and rutaecarpine (10^–1^–10^–5^ M, Solarbio, Beijing, China) were dissolved in dimethyl sulfoxide (DMSO, Sigma-Aldrich, St Louis, MO) before treatment, which was then 1,000 times diluted in the serum-free medium for dosing (10^–4^ and 10^–8^ M). The cells incubated with 0.1% DMSO served as a control.

### Cell Culture

The human glioblastoma cell line U87 (purchased from Cell Resource Center, IBMS, CAMS/PUMC, Beijing, China) and mouse hepatoma cell line CBG2.8D [a stably transfected cell line carrying the DRE-driven luciferase reporter gene, which was established by our own laboratory previously (Zhang S. et al., 2018)] were cultured in Minimum Essential Medium (MEM; Gibco, Paisley, Scotland, United Kingdom) and α-MEM respectively, supplemented with 10% fetal bovine serum (FBS; Corning, NY, United States) and 1% penicillin-streptomycin (Gibco, Paisley, Scotland, United Kingdom). Cells were incubated at 37°C in 95% air, 5% CO_2_.

### Luciferase Reporter Assay

The CBG2.8D is a well-established luciferase reporter-based cell sensor for evaluating the direct activation effect of interest compounds on mouse AhR (Zhang S. et al., 2018; Ma et al., 2019). Due to a lack of a reliable system for evaluating interaction with human AhR. We firstly employed CBG2.8D to reveal if the compounds had a direct effect on AhR activation. Luciferase activity measurement was performed using a luciferase reporter assay system kit (Promega, Madison, WI, United States). The cells were rinsed twice with 50 μL of phosphate-buffered saline after medium removal. Then 50 μL of cell lysis buffer (Promega, Madison, WI, United States) was added into each well following a 15 min shaking at room temperature. Measurement of luciferase activity was carried out using a microplate luminometer (GlomaxMulti Plus, Promega, Madison, WI, United States) with an automatic injection of stabilized luciferase reagent (Promega). The optical signal was collected as relative light unit (RLU) values. All samples were tested in triplicates.

### Gene Expression Knockdown

Cells were transfected with human AhR, IL24 small interfering RNA (siRNA), or non-targeting siRNA (NC) for 24 h before chemical treatments as in the previous study (Liu et al., 2021). The siRNAs were designed and synthesized by SyngenTech Company (Beijing, China). Transfection was conducted using Lipofectamine RNAiMAX transfection reagent (13778500, Invitrogen, Carlsbad, CA, United States) in accordance with the manufacturer’s instructions. After transfection, siRNA and transfection reagent contained medium was replaced with fresh medium, and subsequent analysis was carried out. The final concentration of AhR, IL24, or NC siRNA was 2 × 10***^–^***^8^ M. The sequence of siRNA used in this article was shown in Table 1. To obtain the efficiency of the knockdown, we analyzed the mRNA level of AhR and IL24 after the siRNA transfection as in the previous study (Liu et al., 2021). The knockdown efficiency of AhR and IL24 were 75.2 and 70.1%, respectively, which were consistent with those previously reported (Liu et al., 2021).

**TABLE 1:** siRNA sequence used in this study.

Gene	siRNA sequence
NC	5′-UUC UCC GAA CGU GUC ACC UTT-3
	5′-ACG UGA CAC GUU CGG AGA ATT-3′
AhR NM_001621.5	5′-AGG GAA AGA UGG AUC AAU ATT-3′
	5′-UAU UGA UCC AUC UUU CCC UTT-3′
IL24 NM_001185156.1	5′-GGA GAG CAU UCA AAC AGU UTT-3′
	5′-AAC UCU UUG AAU GCU CUC CTT-3′

### Wound Healing Analysis

According to the description of Cory (2011), a wound-healing experiment was used to evaluate the migration ability of cells. The operation and statistical analysis of wound healing tests were carried out according to the method of Chen et al. (2019). In short, before the cells were inoculated, three parallel lines were drawn at the bottom of each hole of the sterile 6-well plate with a marker pen to locate the wound to be photographed. The cells in the logarithmic growth phase were seeded into the 6-well plate at a density of 2 × 10^5^ cells/ml and placed in the incubator for adherent growth. After the cells cover more than 95% of the plate bottom area, scratch the cell monolayer with the tip of a 1 mL pipette to form a wound. The wound and the parallel line drawn by the marking pen formed a number of crosses, then the wound areas across the lines were selected as the image capturing positions. The floating cells were removed with phosphate buffer and cell-cultured in low serum (1% FBS) medium containing chemicals. Images of the selected wound areas were acquired 0–48 h after the treatments under an inverted microscope (CKX41, Olympus, Japan) with a digital camera (600D, Canon, Japan). For each well, six images were acquired and quantified at each time point. The cell migration was analyzed using Image-Pro Plus 6 software (Media Cybernetics Inc., United States) and the migration distance was calculated by dividing the reduced wound area by the length of the wound in each image. The scale bar in the representative scratch images is 0.5 mm.

### Transwell Migration Analysis

Cell migration and invasion were analyzed using a Transwell permeable support system containing 12 transwell filters (8 μm pore size, Corning, NY, United States). Cells were plated in the upper chamber and treated by the serum-free medium with chemicals or solvent control. And the lower chamber was added into the culture medium (supplemented with 10% FBS) with the same chemical or solvent concentration as the upper chamber. After culturing for 48 h, the cells on the top side of the upper chamber were wiped off by a cotton swab. Cells migrated to the opposite side were fixed with methanol and acetic acid (3:1) for 20 min at room temperature., stained with hematoxylin. Twelve fields of each triplicate filter were counted using an inverted microscope (CKX41, Olympus, Japan). The scale bar in the representative scratch images is 0.1 mm.

### Quantitative Polymerase Chain Reaction

Total RNA was extracted by the GeneJET RNA Purification Kit (Thermo Fisher Scientific Inc., Waltham, MA, United States). Using NanoDrop 2000/2000c Spectrophotometer (Thermo Fisher Scientific Inc., Waltham, MA, United States) to analyze the RNA quality and quantity. cDNA was synthesized using 2 μg total RNA with the RevertAid First Strand cDNA Synthesis Kit (Thermo Fisher Scientific Inc., Waltham, MA, United States). Samples of 20-fold diluted cDNA were subjected to qPCR analysis by using the GoTaq^®^ qPCR Master Mix (Promega, Madison, WI, United States), and the signals were detected by the QuantStudion™6 Flex Real-Time PCR System (Thermo Fisher Scientific Inc., Waltham, MA, United States). Glyceraldehyde phosphate dehydrogenase (GAPDH) expression was not affected by the chemicals and served as an internal control for normalization. Each sample was tested three times. Primers were designed by Primer-BLAST^1^ based on sequences obtained from GenBank^2^ and were synthesized by Sangon Biotech (Shanghai, China). Thermal cycle conditions were as follows: 95°C for 2 min, followed by 40 cycles of denaturation at 95°C for 15 s, annealing at 60°C for 20 s, and extension at 72°C for 20 s. The results were analyzed by the 2***^–^***^ΔΔCT^ value method (Livak and Schmittgen, 2001). The amplification efficiency of the primers was 95–105%. The primer sequences are shown in Table 2.

**TABLE 2:** Primer used for qPCR in this study.

Gene	Primer sequence
AhR NM_001621.5	(F) 5′-CTGAAGTCAACCTCACCAGAAAAAT-3′
	(R) 5′-AAAAACAGTGACTTGTACAGCATAATGA-3′
CYP1B1 NM_000104.4	(F) 5′-GGCCGGTACGTTCTCCAAATC-3′
	(R) 5′-AACGTCATGAGTGCCGTGTGT-3′
IL24 NM_001185156.1	(F) 5′-GCTTCTCTGGAGCCAGGTATCA-3′
	(R) 5′-ACATCTCATTTTCTTGCGAGACG-3′
GAPDH NM_001256799.3	(F) 5′-AGTAGAGGCAGGGATGATG-3′
	(R) 5′-ACAGTCCATGCCATCACTG-3′

### RNA-Seq Analysis and Bioinformatics Analysis

In order to find out the regulation of rutaecarpine on migration-related genes of glioblastoma, the total mRNA of U87 cells was extracted by GeneJET RNA Purification Kit (Thermo Fisher Scientific Inc., Waltham, MA, United States) according to the instruction manual. After verification of integrity and purity, total mRNA was randomly fractured into fragments of approximately 200 bp and transcribed to double-strand DNA with added adaptors. Sequences of resulting DNA products were probed through Illumina NovaSeq 6,000 by Majorbio Ltd, Beijing, China. To identify DEGs (differentially expressed genes) between two different samples, the expression level of each transcript was calculated according to the transcripts per million reads (TPM) method. RNA-Seq by Expectation Maximization (RSEM)^3^ (Li and Dewey, 2011) was used to quantify gene abundances. Essentially, differential expression analysis was performed using the DESeq2/DEGseq/EdgeR with Q value ≤ 0.05, DEGs with | log2FC| > 1 and Q value ≤ 0.05 (DESeq2 or EdgeR)/Q value ≤ 0.001 (DEGseq) were considered to be significantly differentially expressed genes) (Wang et al., 2009; Robinson et al., 2010; Love et al., 2014). Gene ontology (GO) functional analysis was completed by Fisher’s exact test using GOatools Software, and *P* value was adjusted by four tests (Bonferroni, Holm, Sidak and false discovery rate) to control false positive rate when corrected *P*-value (p_fdr) ≤ 0.05 was considered to be significant for GO enrichment. Kyoto Encyclopaedia of Genes and Genomes (KEGG) pathway was analyzed by Fisher’s exact test using KOBAS software. The corrected *P* ≤ 0.05 was considered to be significant for KEGG enrichment. Majorbio bioinformatic analysis cloud computing platform^4^ is used to search and analyze the expression changes of genes. The prediction of putative DRE site(s) on gene promoter is a method for finding candidate genes downstream AhR signaling pathway (Seok et al., 2017). In order to determine whether the expression changes of the migration-related genes of interest are candidate response genes of the AhR pathway, we examined the existence of putative DRE core sequences of 5′-CACGCNA-3′ and 5′-TNGCGTG-3′ on the gene promoters (Li S. et al., 2014). The genetic sequence data of the candidate genes were obtained through GenBank and 2,000-bp DNA fragments upstream of coding sequences of each gene were collected to perform DRE searches.

### Molecular Docking

The three-dimensional structure of AhR (Uniprot AC: P35869) (Ma et al., 2019) were obtained by homology modeling using an online protein structure prediction server, SWISS-MODEL^5^. The chain A of 4zp4.1.B (resolution: 2.36 Å, identity: 27.12%). All the work of molecular docking was done on the Yin Fu cloud computing platform^6^. The initial structures of compounds were constructed and assigned hydrogens by the UCSF Chimera software (Eric et al., 2004), and AMBER ff14SB forcefield and AM1-BCC partial charges (Araz et al., 2000, 2002) were assigned for them. The box center was set at (−87.037, 100.739, −27.384) and the dimensions (27.597, 34.48, 32.672). At last, the affinity was evaluated by the grid-based score using DOCK 6.7 (Lang et al., 2009; Sudipto et al., 2010) to conduct semi-flexible docking with 10,000 different orientations generated. And then the Grid-based score was calculated for each pose. Finally, clustering analysis was performed (RMSD threshold was set 2 Å) to obtain the best-scored poses.

### Statistical Analysis

GraphPad Prism software (version 6, La Jolla, CA, United States) was used for statistical analysis and plotting figures. The values are expressed as the mean ± SEM (*n* = 3), and n represents the number of independent experiments, and each independent sample was analyzed in triplicate. Statistical tests were done by *t*-test while the comparisons involved two groups, one-way or two-way ANOVA with Bonferroni’s test was used in comparisons that involving more than two groups. Statistically significant changes were shown as (*) where *p* < 0.05; (^**^) where *p* < 0.01; and (^***^) where *p* < 0.001.

## Results

### Evodiamine, Dehydroarutaecarpine, and Rutaecarpine Activates AhR Signaling Pathway

**FIGURE 1:**
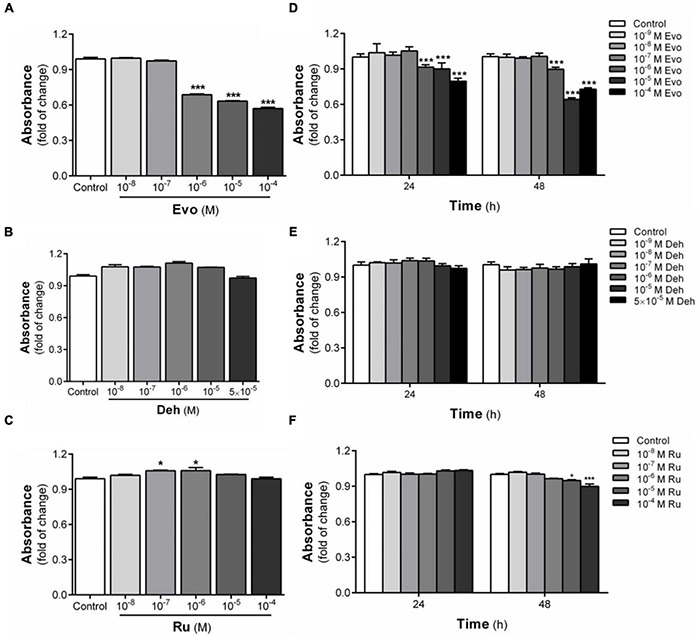
Cytotoxicity of three bioactive substances in Evodia rutaecarpa. **(A)** Effect of evodiamine on cell viability of CBG2.8D cells; **(B)** Effect of dehydroaevodiamine on cell viability of CBG2.8D cells; **(C)** Effect of rutaecarpine on cell viability of CBG2.8D cells; **(D)** Effect of evodiamine on cell viability of U87 cells; **(E)** Effect of dehydroaevodiamine on cell viability of U87 cells; **(F)** Effect of rutaecarpine on cell viability of U87 cells was determined by CCK8 assay. *p < 0.05, ***p < 0.001 compared with control group. All values are fold of control that was treated with the solvent and represent the mean ± SEM (*n* = 3). SEM, standard error of the mean; Evo, evodiamine; Deh, dehydroaevodiamine; Ru, rutaecarpine.

Evodiamine, dehydroaevodiamine, and rutaecarpine are the three main bioactive substances derived from *Evodia rutaecarpa*. To confirm whether evodiamine, dehydroaevodiamine, and rutaecarpine activate the AhR, luciferase reporter assay was performed. Cell viability test showed that evodiamine had a proliferation inhibiting effect on U87 cells. Therefore, in order to avoid interference with the migration assay, evodiamine at 10***^–^***^6^ M was used as the maximum concentration (Figure 1). Compared with the solvent control group, evodiamine, dehydroaevodiamine or rutaecarpine treatment could activate AhR in CBG2.8D cells in a concentration-dependent manner.

**FIGURE 2:**
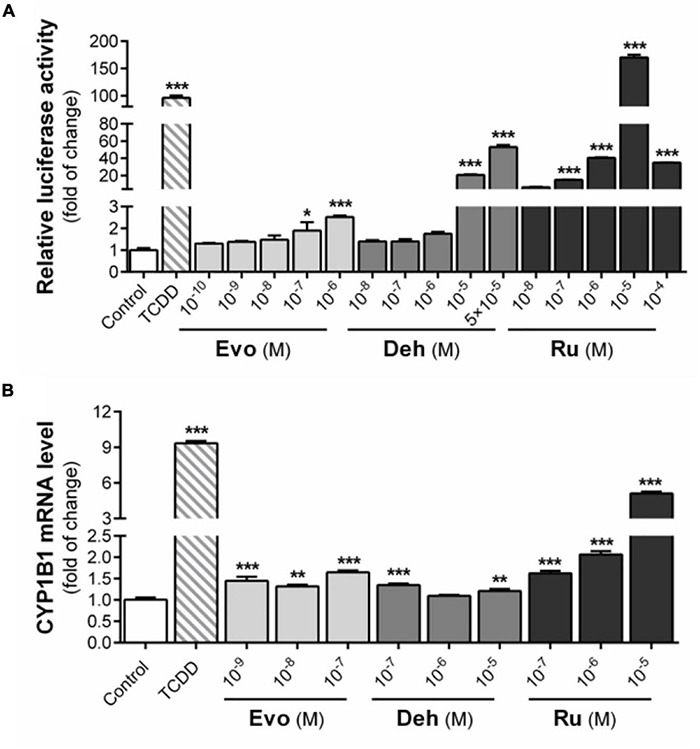
Activation of AhR signaling pathway by three bioactive substances in *Evodia rutaecarpa*. **(A)** Effect of evodiamine, dehydroaevodiamine, and rutaecarpine on activation of AhR after 24 h treatment was determined by luciferase reporter assay in CBG2.8D cells. **(B)** Effect of evodiamine, dehydroaevodiamine, and rutaecarpine on the mRNA expression of CYP1B1 in U87 cells after 48 h treatment were determined by qPCR analysis. GAPDH was used as an internal control for quantification. *p < 0.05, ** *p* < 0.01, ***p < 0.001 compared with control group. All values are fold of control that was treated with the solvent and represent the mean ± SEM (*n* = 3). SEM, standard error of the mean; Evo, evodiamine; Deh, dehydroaevodiamine; Ru, rutaecarpine.

After the 24-h-treatment with 10***^–^***^6^ M evodiamine, dehydroaevodiamine, or rutaecarpine, the luciferase activity was increased by 2.5, 1.8, and 40.5 times, respectively (Figure 2A). At the concentration of 10***^–^***^5^ M, dehydroarutaecarpine and rutaecarpine increased the luciferase activity by 20.8 and 170.2 times, respectively (Figure 2A). The results showed that rutaecarpine (10***^–^***^5^ M) had the strongest activation effect on AhR among three compounds in CBG2.8D cells, and its effect was even higher than the positive control TCDD (10***^–^***^9^ M). However, the activation of AhR by rutaecarpine showed an inverted U-shape concentration-related profile which was due to a decreased induction of rutaecarpine treatment with higher concentrations (10***^–^***^5^ M) (Figure 2A).

To further confirm the effects of these compounds on AhR in human glioblastoma, the expression of the AhR downstream gene CYP1B1 was investigated after being treated with different concentrations of evodiamine, dehydroaevodiamine, and rutaecarpine. In U87 cells, evodiamine and dehydroaevodiamine had a slight effect on the expression of CYP1B1 (Figure 2B). Rutaecarpine had an obvious effect and rutaecarpine at 10***^–^***^5^ M increased the CYP1B1 expression by 5.1 times. These effects were similar to the AhR activation potency in CBG2.8D cells (Figure 2B).

### Effect of Dehydroaevodiamine and Rutaecarpine on the Migration of U87 Cells

**FIGURE 3:**
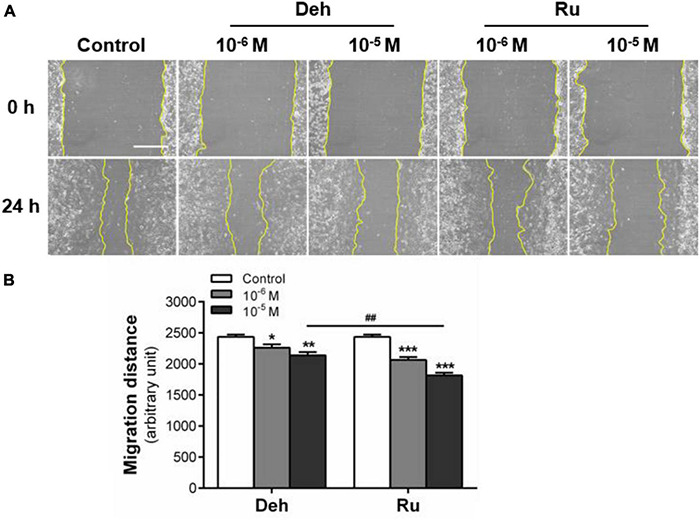
Effect of dehydroaevodiamine and rutaecarpine on the migration of U87 cells. A wound healing assay was used to analyze the effect of compounds on the migration of U87 cells. **(A)** Typical images of wound healing assay were taken at 0 and 24 h about different treatment groups. Scale bar = 0.5 mm. **(B)** Quantitative data graph of wound healing assay. After 24 h treatment with 10***^–^***^6^ and 10***^–^***^5^ M dehydroaevodiamine or rutaecarpine or 0.1% DMSO, the distance of migration was counted. *p < 0.05, **p < 0.01, ***p < 0.001 compared with control groups. ^##^*p* < 0.01, a comparison between Ru (10***^–^***^5^ M) and Deh (10***^–^***^5^ M) treated groups. All values are fold of control that was treated with the solvent and represent the mean ± SEM (*n* = 3). SEM, standard error of the mean; Deh, dehydroaevodiamine; Ru, rutaecarpine.

The effects of dehydroaevodiamine or rutaecarpine treatment on glioblastoma horizontal migration ability were examined by wound healing assay. Typical wound images showed that the wound area of U87 cells increased after dehydroaevodiamine or rutaecarpine treatment (Figure 3A). Quantitative data showed that the inhibition rate of rutaecarpine (10***^–^***^5^ M) on the migration was 25.4% after the 24-h-treatment in U87 cells, while that of dehydroaevodiamine (10***^–^***^5^ M) was 12.1% (Figure 3B). Rutaecarpine inhibited U87 cell migration more potently than dehydroaevodiamine after the same concentration treatment. Therefore, we chose rutaecarpine for the follow-up study.

**FIGURE 4:**
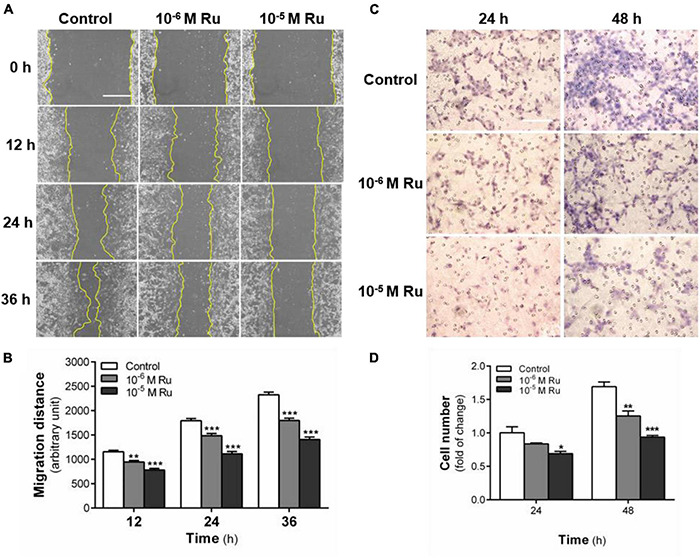
Effect of rutaecarpine on the migration of U87 cells. Wound healing assay and transwell migration analysis were used to analyze U87 cells’ migration ability. **(A)** Typical images of wound healing assay were taken at 0, 12, 24, and 36 h about different treatment groups. Scale bar = 0.5 mm. **(B)** Quantitative data graph of wound healing assay. After 36 h treatment with 10***^–^***^6^ and 10***^–^***^5^ M rutaecarpine or 0.1% DMSO, the distance of migration was counted. **(C)** Typical images of transwell migration analysis were taken at 24 and 48 h about different treatment groups. Scale bar = 0.1 mm. **(D)** Quantitative data graph of transwell migration analysis after 24 h and 48 h treatment with 10***^–^***^6^ and 10***^–^***^5^ M rutaecarpine or 0.1% DMSO, the distance of migration was counted. *p < 0.05, **p < 0.01, ***p < 0.001 compared with control group. All values are fold of control that was treated with the solvent and represent the mean ± SEM (*n* = 3). SEM, standard error of the mean; Ru, rutaecarpine.

Next, we made a more detailed analysis of the inhibitory effect of rutaecarpine on U87 cell migration. We found that the difference between the control group and rutaecarpine group was increased with the prolongation of the drug treatment time (Figure 4A). Quantitative data showed that the cell migration distance was effectively reduced after rutaecarpine treatment at 10***^–^***^6^ or 10***^–^***^5^ M. After 10***^–^***^5^ M rutaecarpine treatment, the migration distance was decreased by 32.5, 37.9, and 39.5% at 12 h, 24 h, and 36 h, respectively (Figure 4B). These data suggested that rutaecarpine can rapidly inhibit the horizontal migration ability of U87 cells at the early stage of drug stimulation. Such inhibitory effect of rutaecarpine is sustained with time but with decreased inhibition rates. Then, transwell migration analysis was also used to verify the effect of rutaecarpine on the migration of U87 cells, and we got similar results with that of wound healing analysis (Figures 4C,D).

### AhR Signaling Pathway Mediates the Inhibition of Rutaecarpine on U87 Cells Migration

**FIGURE 5:**
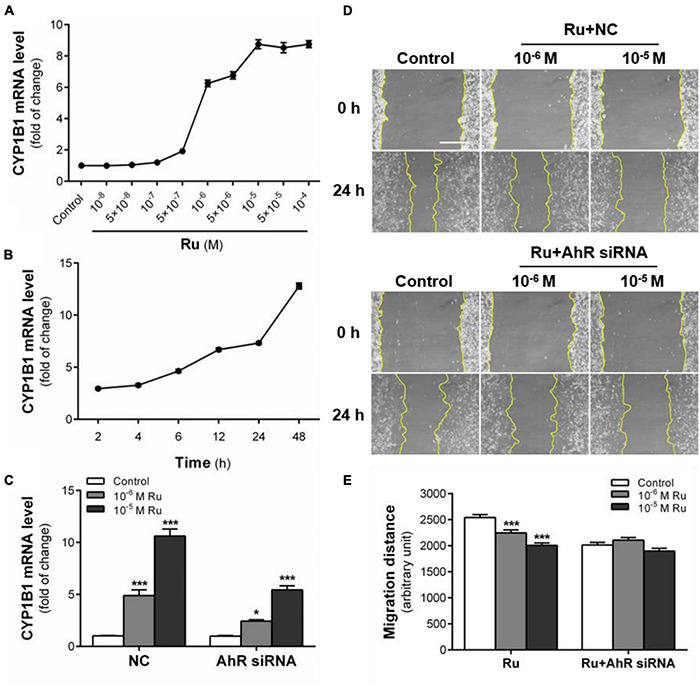
AhR signaling pathway mediates the inhibition of rutaecarpine on U87 cells migration. **(A)** Dose relationship between rutaecarpine with AhR signaling pathway activation after 48 h treatment with 10***^–^***^8^–10***^–^***^4^ M rutaecarpine or 0.1% DMSO. **(B)** Time relationship of rutaecarpine with AhR signaling pathway activation after 2, 4, 6, 12 24, 48 h treatment with 10***^–^***^4^ M rutaecarpine. **(C)** After 48 h treatment with rutaecarpine or 0.1% DMSO together with or without AhR siRNA pre-treatment, the mRNA expression of CYP1B1 in U87 cells. The mRNA expression of CYP1B1 in U87 cells was determined by qPCR analysis. GAPDH was used as an internal control for quantification. A wound healing assay was used to analyze the effect of AhR signaling pathway activation on U87 cells migration. AhR mRNA interference was treated as indicated. **(D)** Typical images of wound healing assay were taken at 0 and 24 h about different treatment groups. Scale bar = 0.5 mm. **(E)** Quantitative data graph of wound healing assay. After 24 h treatment with 10***^–^***^6^ and 10***^–^***^5^ M rutaecarpine or 0.1% DMSO, the distance of migration was counted. *p < 0.05, ***p < 0.001 compared with control group. All values are fold of control that was treated with the solvent and represent the mean ± SEM (*n* = 3). SEM, standard error of the mean; Ru, rutaecarpine; NC, non-targeted siRNA.

Rutaecarpine showed a time and concentration-dependent inductive effect on CYP1B1 mRNA expression (Figures 5A,B). After 48 h exposure, 10***^–^***^6^ and 10***^–^***^5^ M rutaecarpine increased the expression of CYP1B1 by 6.3 and 8.8 times, respectively. When the concentration was higher than 10***^–^***^5^ M, the activation of AhR reached a plateau (Figure 5A). With regard to the time effect, we found that the induction of CYP1B1 was also increased over time, even after 48 h of the rutaecarpine treatment (Figure 5B). The data suggested that a long-lasting effect of rutaecarpine on AhR activation in U87 cells. In addition, after the AhR knockdown, CYP1B1 induction caused by rutaecarpine was decreased by ∼50% (from 10.6-fold to 5.4-fold) (Figure 5C). Considering ∼70% efficiency of AhR siRNA this result indicated the CYP1B1 induction was mostly dependent on AhR in U87 cells.

Next, we explored the role of AhR in rutaecarpine-induced migration inhibition. After the addition of non-targeted siRNA (NC group), the inhibition of rutaecarpine on cell migration did not change (Figure 5D). Similar to other AhR agonists from the literature, we found that the migration ability of U87 cells was decreased after AhR knockdown. In the AhR siRNA treated group, rutaecarpine lost mostly all the inhibitory effects on the cell migration (Figure 5E). Even in the 10***^–^***^6^ M rutaecarpine treatment group, only a slight but not significant decrease in migration distance was obtained after the AhR knockdown (Figure 5E). These results suggest that rutaecarpine-induced migration inhibition is mostly in an AhR-dependent manner.

### Simulate Interactions Between Rutaecarpine and AhR

**FIGURE 6:**
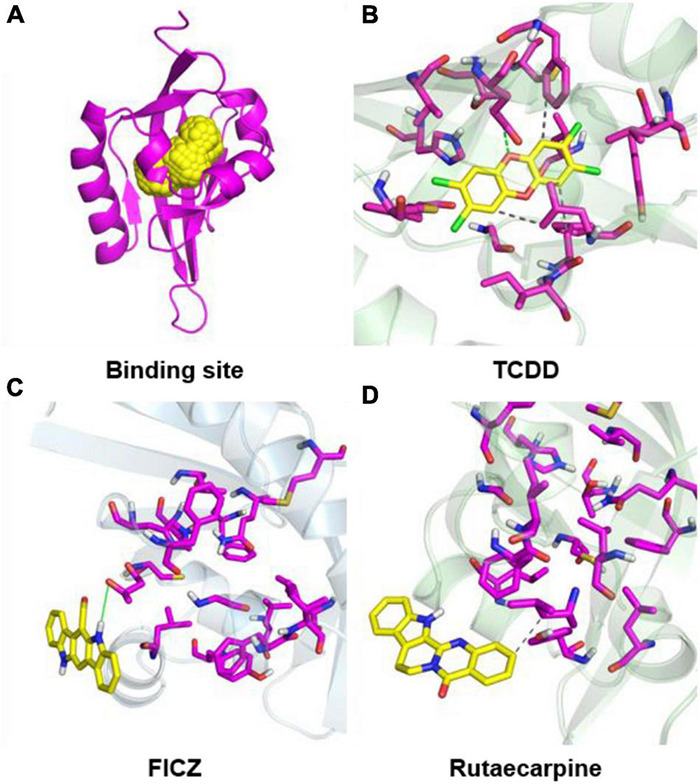
Molecular docking configurations of TCDD, FICZ, and rutaecarpine with AhR. **(A)** AhR_HUMAN binding site map. The secondary structure of the protein was shown by magenta bands and the binding sites were shown by yellow spheres. **(B)** Docking of TCDD into the AhR binding pocket. **(C)** Docking of FICZ with the AhR protein model. **(D)** Docking of rutaecarpine with the AhR protein model.

In addition to the experimental studies, the potential capabilities of rutaecarpine in binding to AhR were evaluated computationally. The software analysis shows that the quality of homologous modeling is qualified and can be used for the molecular docking model (Supplementary Figure 1). The interaction modes between rutaecarpine and AhR were simulated by DOCK 6.7. Well-known AhR ligands TCDD and FICZ were used as controls. It has been reported that TCDD binds to AhR Human residues Cys300, Leu308, Tyr310, Leu315, Phe324, Ile325, Cys333, His337, Met348, and Leu353. Based on this, the binding site was constructed and shown in the yellow sphere in Figure 6A. Three compounds had a high affinity for AhR, and TCDD was successfully docked into the binding pocket (Figure 6B). FICZ and rutaecarpine are five-membered rings with large structures and are difficult to exist in the narrow pocket, which can only bind to the residues outside the predicted binding pocket (Figures 6C,D). The score value indicated the interaction activity between compounds and AhR, with smaller score values indicating the less energy required for intermolecular binding and the better the interaction activity. The docking scoring results showed that the affinity of the compounds was almost contributed by van der Waals force, the electrostatic force and internal repulsive are negligible (Table 3). Different spatial pose of molecules has different binding abilities with the receptor. The energy of TCDD, FICZ, and rutaecarpine binding to AhR were −38.92, −26.48, and −21.82 kcal/mol, respectively, under the best combination pose (Table 3).

**TABLE 3:** Binding energy (kcal/mol) and molecular docking scoring of the AhR with target chemicals.

Compound	Pose	Score	Vdw_energy	Es_energy	Repulsive
TCDD	1	−38.92	−37.76	−1.15	0
	2	−38.90	−37.81	−1.09	0
	3	−38.83	−37.70	−1.13	0
	4	−38.62	−37.55	−1.06	0
	5	−9.27	−8.84	−0.42	0
	6	−8.69	−8.24	−0.45	0
	7	−4.86	−5.23	0.36	0
	8	−4.60	−5.04	0.44	0
FICZ	1	−26.48	−27.04	0.55	7.29
	2	−18.71	−19.49	0.77	1.86
	3	−16.89	−17.13	0.24	1.93
	4	−14.67	−14.83	0.16	7.29
Rutaecarpine	1	−21.82	−21.37	−0.45	0
	2	−17.74	−17.79	0.05	0
	3	−17.66	−17.52	−0.13	0
	4	−17.19	−16.61	−0.57	0
	5	−16.02	−15.93	−0.09	0
	6	−12.35	−12.43	0.07	0
	7	−10.09	−10.33	0.24	0

*Score, total score of molecular docking (kcal/mol); Vdw_energy, Van der Waals energy (kcal/mol); Es_energy, electrostatic energy between molecules (kcal/mol); Repulsive, internal energy repulsive (kcal/mol).*

### Rutaecarpine Inhibits Glioblastoma Migration Predominantly Through the AhR-IL24 Axis

**FIGURE 7:**
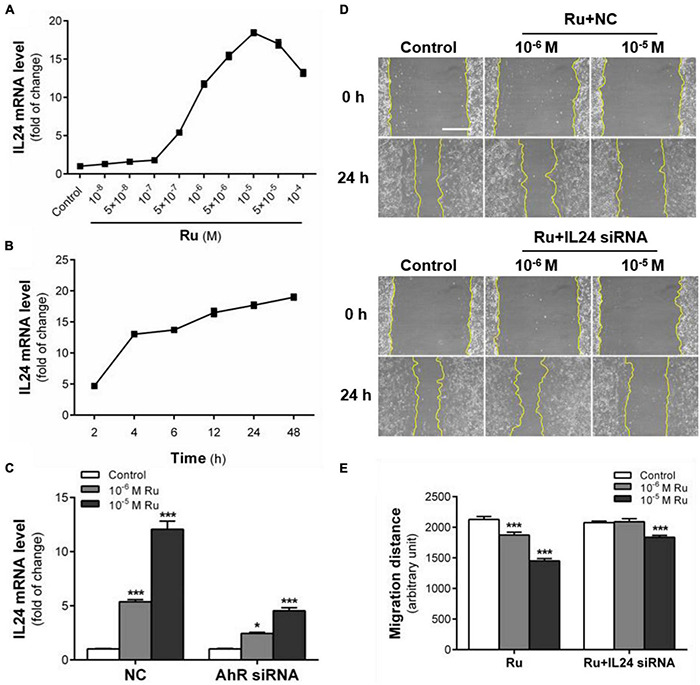
IL 24 mediates the inhibitory effect of rutaecarpine on migration. **(A)** Dose relationship between rutaecarpine with IL24 expression after 48 h treatment with 10***^–^***^8^–10***^–^***^4^ M rutaecarpine or 0.1% DMSO. **(B)** Time relationship of rutaecarpine with IL24 expression after 2, 4, 6, 12, 24, 48 h treatment with 10***^–^***^4^ M rutaecarpine. **(C)** Effect of rutaecarpine 48 h treatment on IL24 expression before and after AhR knockdown. The mRNA expression of IL24 in U87 cells was determined by qPCR analysis. A wound healing assay was used to analyze the effect of IL24 on U87 cells migration. IL24 mRNA interference was treated as indicated. **(D)** Typical images of wound healing assay were taken at 0 and 24 h about different treatment groups. Scale bar = 0.5 mm. **(E)** Quantitative data graph of wound healing assay. After 24 h treatment with 10***^–^***^6^ and 10***^–^***^5^ M rutaecarpine or 0.1% DMSO, the distance of migration was counted. *p < 0.05, ***p < 0.001 compared with control group. All values are fold of control that was treated with the solvent and represent the mean ± SEM (*n* = 3). SEM, standard error of the mean; Ru, rutaecarpine.

With the newly reported role of the AhR-IL24 axis in TCDD-induced migration inhibition of U87 cells, we further explored the effect of rutaecarpine on IL24 expression. After exposure to rutaecarpine at different concentrations, the expression of IL24 mRNA was gradually increased and reached the maximum induction at 10***^–^***^5^ M, which was about 18.4 times compared with the control group. However, the induction rate of IL24 began to decline in treatment with higher concentrations (Figure 7A). Like rutaecarpine-induced effect on CYP1B1 expression, 10***^–^***^5^ M rutaecarpine increased the expression of IL24 over time. After 48 h treatment, the mRNA level of IL24 was 19.1 times higher than that of the control group and showed an incline tendency (Figure 7B). The IL24 induction rate by rutaecarpine was reduced by 62.5% after the AhR knockdown (Figure 7C). Considering ∼70% efficiency of the AhR siRNA, we speculated the induction of IL24 caused by rutaecarpine was mostly AhR-dependent. Furthermore, we found that rutaecarpine (10***^–^***^5^ M) lost the ability to inhibit the cell migration by ∼20.0% (the inhibition changed from 32.5 to 12.5%) after the IL24 knockdown (IL24 siRNA efficiency was ∼70%), suggesting that the rutaecarpine effect on migration at least partially depends on the induction of IL24 in U87 cells (Figures 7D,E). This finding differs from the dominant role of AhR in the rutaecarpine-induced migration inhibition, which suggests that there may be other AhR responsive genes involved in rutaecarpine-induced migration inhibitory except for IL24.

### Effects of Rutaecarpine on Tumor and Cell Migration-Related Genes in Glioblastoma

**FIGURE 8:**
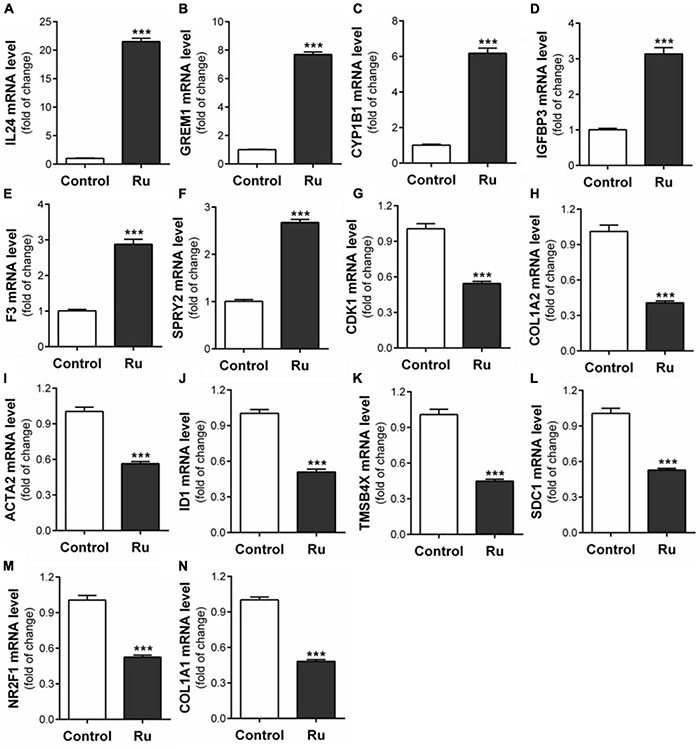
Verification of fourteen migration related genes in RNA-seq results. After U87 cells were treated with rutaecarpine for 48 h, the mRNA expressions of **(A)** IL24, **(B)** GREM1, **(C)** CYP1B1, **(D)** IGFBP3, **(E)** F3, **(F)** SPRY2, **(G)** CDK1, **(H)** COL1A2, **(I)** ACTA2, **(J)** ID1, **(K)** TMSBAX, **(L)** SDC1, **(M)** NR2F1, and **(N)** COL1A1 were determined by qPCR analysis. GAPDH was used as an internal control for quantification. ***p < 0.001 compared with control group. All values are fold of control that was treated with the solvent and represent the mean ± SEM (*n* = 3). SEM, standard error of the mean; Ru, rutaecarpine.

In order to find other potential migration-related genes involved in rutaecarpine-induced migration inhibition that are responsive to AhR activation, RNA-Seq analysis, and putative DRE analysis were employed. There were 13,743 commonly identified genes in the control group and rutaecarpine treatment group based on the expression analysis. Among them, 952 genes were defined as DEGs based on the aforementioned criteria, including 404 up-regulated genes and 548 down-regulated genes (Supplementary Figure 2). Firstly, by using “migration” as the keyword to search genes according to the functional annotation screening tool, we obtained 119 database-identified migration-related genes from the 952 DEGs. After screening out the low expression (TPM < 2) genes, we further identified 35 up-regulated and 50 down-regulated migration-related genes according to their literature reported functions (Supplementary Tables 1, 2). On the other hand, the 952 DEGs were clustered based on KEGG analysis, including one cancer-related pathway as “cancer overview” (Supplementary Figure 3). We found that there are 20 genes among the 85 migration-related genes belonging to the cancer-related cluster. Among the 20 candidate genes, 14 representative genes were validated using qPCR analysis, including IL24, GREM1, CYP1B1, IGFBP3, F3, SPRY2, CDK1, COL1A2, ACTA2, ID1, TMSB4X, SDC1, NR2F1, and COL1A1 (Table 4). By qPCR analysis, we found the alterations in the gene expression were consistent with that of RNA-seq, in which *t*-test was used in the comparison of each gene (Figure 8). Moreover, prediction of putative DRE site(s) was performed on these 14 validated gene promoters to evaluate their potential for responding to AhR activation. We found all expression-validated cancer and migration-related genes had at least one putative DRE(s) in their promoters, especially CYP1B1, ID1, and SDC1, which had 8, 5, and 6 putative DRE sites, respectively (Table 4). One putative DRE site was found in the IL24 promoter, which has been identified to mediate TCDD-induced upregulation by chromatin immunoprecipitation analysis previously (Liu et al., 2021).

**TABLE 4:** Statistics of DRE sequences in 14 migration-related gene promoters.

Gene name	Gene ID	DRE number	Fold change	Function
IL24	11,009	1	25.57	Inhibit
GREM1	26,585	1	7.00	Inhibit
CYP1B1	1,545	8	5.36	Inhibit
IGFBP3	3,486	3	2.46	Inhibit
F3	2,152	1	2.35	Inhibit
SPRY2	10,253	3	2.60	Inhibit
CDK1	983	3	0.41	Promote
COL1A2	1,278	3	0.39	Promote
ACTA2	59	2	0.40	Promote
ID1	3,397	5	0.23	Promote
TMSB4X	7,114	2	0.34	Promote
SDC1	6,382	6	0.41	Promote
NR2F1	7,025	2	0.45	Promote
COL1A1	1,227	2	0.41	Promote

## Discussion

Evodiamine, dehydroevodiamine, and rutaecarpine are three major bioactive components of *Evodia rutaecarpine* (Zhang Y. et al., 2018; Zhang et al., 2020). Their effects on cardiovascular protection, hepatic lipid metabolism regulation, liver injury protection, degenerative neurological diseases improvement, and acute pancreatitis protection have been fully recorded (Yan et al., 2018; Tian et al., 2019; Li et al., 2020; Zhao B. et al., 2020). Recently, some studies have found that evodiamine and rutaecarpine also have anti-tumor activity *in vivo* and *in vitro* experiments, which can induce apoptosis and inhibit the proliferation, invasion, and metastasis of a variety of tumors (Ogasawara et al., 2001; Jiang and Hu, 2009; Yang et al., 2017). However, few studies about the effect of these three bioactive components on glioblastoma was reported. In our research, we found that rutaecarpine, but not evodiamine and dehydroevodiamine, can upregulate the expression of IL24 by activating the AhR signaling pathway, thereby inhibiting the migration of glioblastoma.

Chemotherapy is widely used in the clinical treatment of cancer (MacDonald, 2009). Cancer chemotherapy drugs such as temozolomide and semustine have strong toxicity, which can kill tumor cells and damage normal cells at the same time, leading to a high incidence of complications (Nadége et al., 2002). What makes it worse is that tumor cells could be resistant to most chemotherapy drugs and targeted drugs during long-term chemotherapy. The drug resistance is intrinsic or acquired which limits the effectiveness of chemotherapy and increases the mortality of patients (Longley and Johnston, 2005; Brian et al., 2012). In recent years, some new drugs and adjuvant therapies have been reported. For example, Lakshmi et al. (2019) loaded paclitaxel onto multifunctional nanoparticles for the targeted treatment of glioblastoma. Chemotherapy combined with curcumin or other natural compounds can effectively improve the drug sensitivity of glioblastoma and enhance the tumor inhibitory effect of TMZ (Yin et al., 2014; Liu et al., 2020). Compared with the widely used aggressive cancer chemotherapy drugs, the main advantage of natural compounds such as paclitaxel and curcumin is their low biological toxicity. Meanwhile, a large number of *in vitro* experiments have confirmed that they have potential anti-tumor effects on a series of malignant tumors (Wang et al., 2020). Many natural compounds can resist drug resistance by regulating drug-resistant proteins and targeting non-apoptotic cell death (Yuan et al., 2017). In addition, many natural compounds have good tolerance to patients and will not cause toxic effects even in high doses, thus, they have been put into the clinical test of cancer therapy (Packer et al., 1997; Rejhová et al., 2018; Shroff et al., 2019). Ogasawara et al. (2001) studied the effects of 75 natural compounds on the migration and proliferation of colon cancer 26-L5 cells *in vitro*. They found that rutaecarpine (100 μg/mL) can inhibit the migration of tumor cells by about 30–40% without cell cytotoxic effects, which is the most effective one in all selected compounds. These findings suggest that rutaecarpine might be a potential candidate for developing new anti-tumor drug(s) (Ogasawara et al., 2001).

Although rutaecarpine exhibits promising anti-tumor effects in certain types of tumors, including breast and liver cancer (Guo et al., 2016), its anti-tumor action mechanism is still not clear. In this study, we found new evidence showing that rutaecarpine also has inhibitory effects on human glioblastoma cell migration, and the activation of AhR which triggers the AhR-IL24 anti-migratory axis is an important action mechanism for the anti-migratory effects. To our knowledge, these results provide new evidence for rutaecarpine’s anti-tumor effects and underlining mechanisms. Furthermore, the activation of AhR by rutaecarpine leads to the induction of the AhR-IL24 anti-migration axis, which may be an important mechanism for its anti-glioblastoma effects. Regarding the interaction of rutaecarpine with AhR, we provide both theoretical and experimental evidence demonstrating that rutaecarpine is a promising natural stimulant of mouse and human AhR. The results of molecular docking simulation showed that rutaecarpine was capable to interact with AhR, which is consistent with the molecular docking results of Zhang Y. et al. (2018). The binding mode of rutaecarpine is through interaction with residues outside the binding site instead of getting into the binding pocket of AhR. According to our data and others (Zhang Y. et al., 2018), a similar interaction model with AhR has been found for the well-known AhR agonist FICZ, which has a large spatial structure as that of rutaecarpine. We also provided experimental evidence to support the interaction between rutaecarpine and AhR in mouse hepatoma cells using the well-established DRE-driven reporter gene assay (Zhang S. et al., 2018), as well as in human glioblastoma cells *via* determination of the classical AhR responsive gene CYP1B1. In line with our findings, rutaecarpine has been found to AhR-dependently induce the downstream gene expression in human hepatoma cells and keratinocytes (Han et al., 2009; Haarmann-Stemmann et al., 2010). These findings suggest that the activation of AhR by rutaecarpine is not solely occurring in the glioblastoma cells. Compared with other structurally related compounds, such as evodiamine and dehydrogenated evodiamine, we found rutaecarpine has the strongest AhR activation effects. Zhang Y. et al. (2018) also reported that rutaecarpine has a better activation effect than the other two kinds of evodia-derived compounds, evodiamine, and dehydroevodiamine. With the promising interaction with human AhR, we further demonstrated that rutaecarpine exerted its anti-migratory function in an AhR-dependent manner by using the AhR knockdown study. The responsive gene downstream AhR pathway that participates in the anti-migratory effect of rutaecarpine is another key issue to reveal the action mechanism. Recently, the regulatory role of the AhR-IL24 axis in the anti-migratory effect of TCDD on glioblastoma, breast cancer, and lung cancer cells has been documented (Liu et al., 2021). Based on the present results and known effects of IL24 on tumors, we propose an important role of IL24 in the effect of rutaecarpine and emphasize the notion that IL24 was one of the major downstream genes of the AhR pathway responsible for the inhibition of U87 migration. With its AhR activity in multiple human cancer cells, rutaecarpine may cause AhR-dependent regulation on cell migration in other cancer cell types, which is worthy of further investigations. As supportive evidence for this hypothesis, it has been demonstrated that apart from the U87 human glioblastoma cell, the TCDD-induced AhR-IL24 axis plays role in the anti-migratory effects in human breast cancer and lung cancer cells (Liu et al., 2021).

In this study, we found dose-dependent induction of IL24 by rutaecarpine treatment in U87 cells which was mostly due to the activation of AhR. Different from the case of TCDD, we found that the inhibition of cell migration caused by rutaecarpine was reversed by ∼60% after the IL24 knockdown. Considering the ∼70% efficiency of IL24 siRNA, we proposed a possibility that other migration-related AhR downstream genes might be involved. With the bioinformatic analysis, we obtained 13 more candidate genes besides IL24 from the RNA-seq data, which might serve as supplements of IL24 that contribute to the AhR-dependent mechanism for the anti-migration effects of rutaecarpine. However, the detailed regulation mechanism and interaction network for these genes still need further investigation.

IL24 is a pleiotropic immune cytokine in the IL10 family, as well as a broad-spectrum tumor suppressor (Deng et al., 2021), which can induce apoptosis, inhibit angiogenesis, block metastasis and increase the sensitivity of radiotherapy and chemotherapy without affecting the normal cells (Mohammad et al., 2019). Therefore, many scientists regard IL24 as a promising molecular therapy for cancers and apply it in the phase I clinical trial (Cunningham et al., 2005). The anti-tumor activity of IL24 is mainly attributed to endogenous gene expression (Mohammad et al., 2019). The evidence shows that overexpression of IL24 has been found to inhibit the migration of pancreatic cancer, lung cancer, neuroblastoma, and hepatocellular carcinoma cells (Zhuo et al., 2013; Han et al., 2015; Janani et al., 2015; Jia et al., 2016). Emerging evidence has shown the relationship between IL24 and AhR. According to gene expression profile analysis, Luo et al. (2017) found that IL24 was the most highly inducible gene among the cytokines regulated by AhR after activating AhR in the lung adenocarcinoma cell line CL5 with benzo[a]pyrene (BaP), and it could be blocked by AhR antagonists. Liu et al. (2015) identified the AhR-dependent transcriptional upregulation of the IL24 gene in normal human chorionic villi. TCDD, FICZ, and tapinarof also can activate the expression of IL24 through AhR in various cell types (Vu et al., 2020). Besides IL24 and CYP1B1, we also found other migration-related genes that may be regulated by AhR through transcriptome sequencing and DRE analysis. They may participate in the regulation of U87 cell migration and serve as potential downstream responsive gene(s) of the AhR pathway in human glioblastoma cells. It can also explain why the inhibition of rutaecarpine on cell migration cannot be completely blocked after IL24 knockdown.

AhR activity is necessary to maintain normal cell functioning. It participates in a variety of physiological activities including cell adhesion and migration which are of great importance to tumor progression. However, the effects of AhR on cell migration are diverse in different cell types, so it is difficult to define whether AhR is a tumor promoter or a suppressor (Pedro, 2010). For example, Contador-Troca et al. (2013) found that AhR in melanoma blocks the growth and metastasis of melanoma cells, while it supports the development of melanoma when AhR was expressed in the stroma, so they proposed that AhR might be involved in the tumor-stroma interaction in melanoma. In glioma or glioblastoma, AhR expression has been positively related to malignancy and tumor promotion (Silginer et al., 2016; Anthony et al., 2018). We previously proposed that basal level activation of AhR can maintain the high motility of human glioblastoma cells, and the exogenous AhR ligands, such as TCDD and rutaecarpine, can competitively bind to AhR to weaken or eliminate the effect of the endogenous ligands and lead to the inhibiting effect on cell migration ability (Liu et al., 2021). However, the endogenous ligand(s) responsible for the basal activation of AhR remains a mystery in glioblastoma cells. On the other hand, in terms of the constitutive expression of AhR, we also demonstrated that glioblastoma cells with higher constitutive AhR expression had higher migration ability (Liu et al., 2021). Such a positive correlation between the expression of AhR and the migration ability of glioblastoma cells was also found upon the activation AhR signaling pathway, in which the cell migration ability was decreased with a reduction in AhR mRNA expression (Liu et al., 2021). Consistent with these findings, in AhR knockout cells, no matter with or without AhR ligands, the migration ability was reduced compared to NC groups (Figure 5; Liu et al., 2021). Therefore, based on our data and others, the constitutive expression of AhR has multiple tumors promoting effects in certain glioma and glioblastoma cells. AhR exhibits diverse regulatory roles in different types of tumors or upon activation by diverse ligands. It has been reported that TCDD, BAP, pyocyanin, indoxyl sulfate, and other agonists can induce the migration and invasion of human breast cancer MDA-MB-231, Hs578T, SUM149 cells, oral squamous cell carcinoma *via* activating the AhR signaling pathway (Stanford et al., 2016; Narasimhan et al., 2018; Shadboorestan et al., 2019). While agonists 2-(1H-indole-3-carbonyl)-thiazole-4-carboxylic acid methyl ester (ITE), omeprazole, glyceolin I, glyceolin II can target AhR to limit the expression of migration regulatory factors MYH9, CXCL12, CXCR4, MMP9, CDH2, CCL2 and inhibit the migration and proliferation of glioblastoma or human breast cancer cells (Hall et al., 2010; Pham et al., 2020; Zhao L. et al., 2020). Notably, the influences of AhR on tumors are tissue or ligand-specific. However, in U87 cells, activation of AhR by TCDD, FICZ or rutaecarpine, resulted in similar migration inhibition, which might rely on the similar mechanism upon the different AhR activation. However, the AhR expression is variable in different glioblastoma cells (Jin et al., 2019). The AhR expression in U87 cells might be different from other glioblastoma cells, such as U-251 based on the database information^7^ and A172 based on our own data (data not shown), in which there is almost no AhR expression. Therefore, the AhR-dependent mechanism reported in this study may not exist in glioblastoma cells that express very little or no AhR. Given the importance of AhR in rutaecarpine-induced effects in U87 cells, if this compound has similar anti-migratory effects in the glioblastoma cells expressing less AhR is still unclear. We speculate that is most likely beyond the AhR-dependent issue. Based on the relatively high expression of AhR in highly malignant glioblastoma from the histopathology study (Gramatzki et al., 2009), we consider U87 as a representative of such high-level AhR-expressing malignant glioblastoma and emphasize the plausibility of considering rutaecarpine as a potential anti-migratory compound in the malignant glioblastoma cells with high-level AhR expression. In this context, AhR and IL24 might be used as composite targets for screening anti-tumor migration compounds in there.

In conclusion, we found that rutaecarpine is a natural compound with AhR activation activity, which can inhibit the migration of U87 human glioblastoma cells by activating the AhR signaling pathway and inducing the expression of downstream tumor suppressor IL24. At the same time, rutaecarpine can also regulate other migration and tumor-related gene expression which might be potentially regulated by AhR. As a promising naturally derived AhR agonist, rutaecarpine was proposed as a potential candidate for developing drugs against glioblastoma migration, particularly those with high AhR expression. Meanwhile, we propose AhR and IL24 as composite targets for screening anti-glioblastoma migration compounds.

## Data Availability Statement

The datasets presented in this study can be found in online repositories. The names of the repository/repositories and accession number(s) can be found below: https://www.ncbi.nlm.nih.gov/geo/query/acc.cgi?acc=GSE183606.

## Author Contributions

YL was responsible for experimental design, experimental operation, data curation and writing—original draft preparation. YC and RZ were responsible for the experimental operation and manuscript preparation. LX, HX, and BZ were jointly responsible for conceptualization, supervision, and writing—review and editing. All authors contributed to the article and approved the submitted version.

## Conflict of Interest

The authors declare that the research was conducted in the absence of any commercial or financial relationships that could be construed as a potential conflict of interest.

## Publisher’s Note

All claims expressed in this article are solely those of the authors and do not necessarily represent those of their affiliated organizations, or those of the publisher, the editors and the reviewers. Any product that may be evaluated in this article, or claim that may be made by its manufacturer, is not guaranteed or endorsed by the publisher.

## Footnotes

1
https://www.ncbi.nlm.nih.gov/tools/primer-blast/

2
http://www.ncbi.nlm.nih.gov/genbank/

3
http://deweylab.biostat.wisc.edu/rsem/

4
http://www.majorbio.com/

5
http://swissmodel.expasy.org/

6
http://cloud.yinfotek.com/

7
https://www.proteinatlas.org/ENSG00000106546-AHR/cell
