# Supplementary material for: Rutaecarpine Inhibits U87 Glioblastoma Cell Migration by Activating the Aryl Hydrocarbon Receptor Signaling Pathway
Source: Front Mol Neurosci. 2021 Dec 9;14:765712. doi: 10.3389/fnmol.2021.765712 (PMC8696176; doi:10.3389/fnmol.2021.765712)
Supplement: Supplementary file 1 [file Data_Sheet_1.pdf]

Supplementary Material

1 Supplementary Figures and Tables

1.1 Supplementary Figures

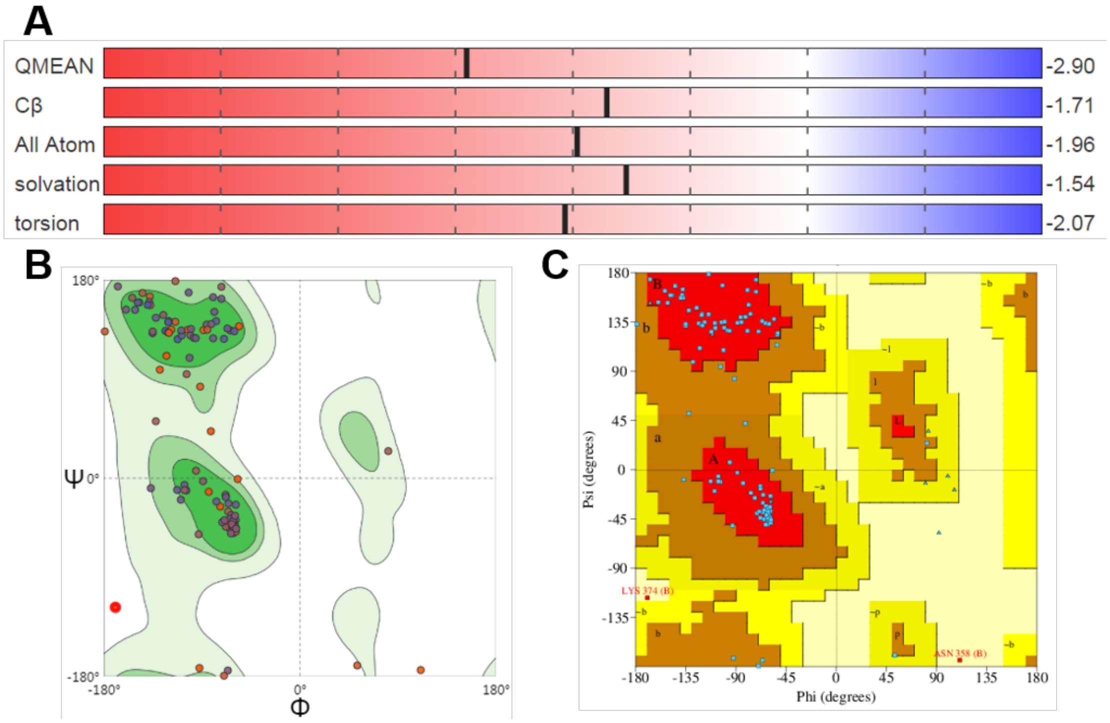

Supplementary Figure 1. Quality evaluation of homologous modeling. (A) The quality of homologous modeling was evaluated by online analysis tool SWISS-MODEL. (B) (C) Ramachandran diagram of protein structure.

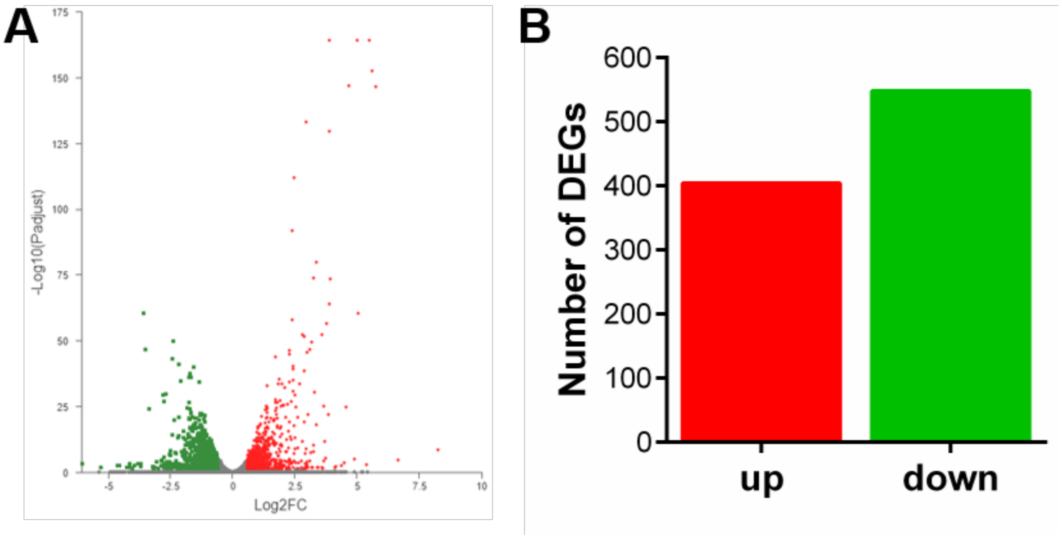

Supplementary Figure 2. Statistics of differentially expressed genes. (A) Differentially expressed genes (DEGs) between different treatment groups. The horizontal axis indicates expression changes (log) of the genes in different treatment groups while the vertical axis shows the differences of gene expression. Splashes were for different genes, among which grey dots were genes with no significant discrepancy, red dots were genes significantly up-regulated and green dots were significantly down-regulated genes. (B) Number of differentially expressed genes (DEGs) at adjusted p ≤ 0.05, and |log2fc| fold change ≥ 1: Within and between genotype comparisons for U87 cells with and without rutaecarpine treatment.

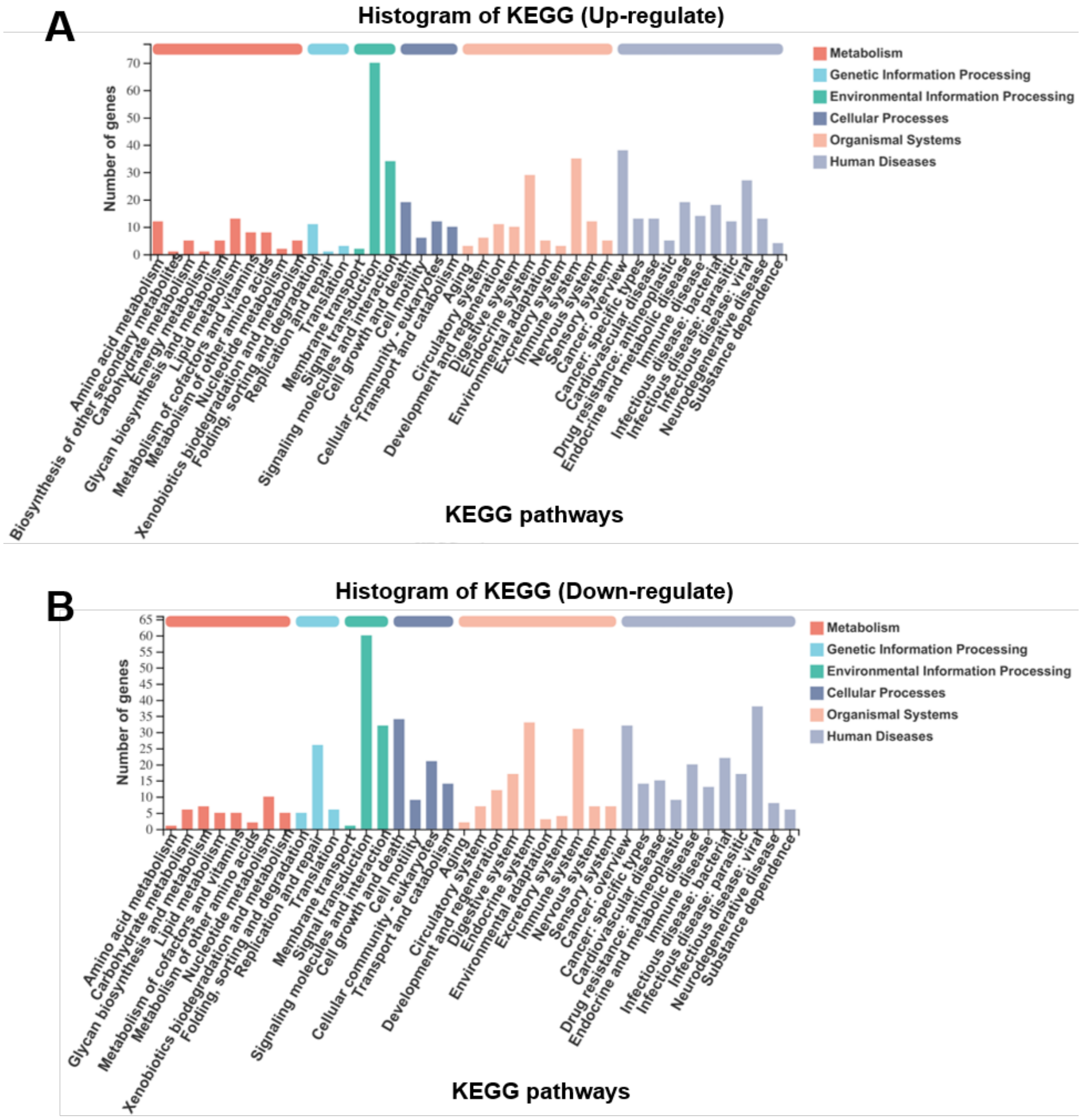

Supplementary Figure 3. KEGG classification and pathway enrichment of DEGs.

1.2 Supplementary Tables

Supplementary Table 1. Up-regulated differentially expressed genes associated with migration.

mRNAs	Fold change	Function	mRNAs	Fold change	Function
IL1β	32.29	Promote[1]	SLC3A2	2.83	Promote[17]
IL24	25.57	Inhibit[2]	SSH1	2.77	Promote[18]
PTGS2*	15.03	Promote[3]	WNT5A*	2.70	Inhibit/Promote[19]
CCL20	8.61	Promote[4]	SPRY2*	2.60	Inhibit[20]
GREM1	7.00	Inhibit[5]	LPXN	2.58	Promote[21]
THBS1*	6.16	Promote[6]	ICAM1	2.51	Promote[22]

CYP1B1*	5.36	Inhibit[7]	IGFBP3*	2.46	Inhibit[23]
FGF2*	4.92	Promote[8]	F3	2.35	Inhibit[24]
CARD10	3.96	Promote[9]	SLC7A5	2.28	Promote[25]
AL390719.1	3.77	/	SFRP2	2.28	Inhibit[26]
HSPA5	3.75	Promote[10]	ITGA2*	2.21	Promote[27]
SLC7A11	3.63	Inhibit[11]	CEMIP2	2.16	Promote[28]
MMP1*	3.50	Promote[12]	MMP14	2.13	Promote[29]
HAS2	3.39	Promote[13]	NFE2L2*	2.11	/
NTN4	3.14	Inhibit[14]	BEX4	2.06	/
ITGA11	3.13	Promote[15]	IRS2*	2.05	Promote[30]
SRGAP3	3.00	/	ITGB3*	2.05	Promote[31]
ITGB8	2.87	Promote[16]			

* is the genes in the intersection of the two gene sets described as result section 3.5. The references in this table are shown in the end of this supporting information.

Supplementary Table 2. Down-regulated differentially expressed genes associated with migration.

mRNAs	Fold change	Function	mRNAs	Fold change	Function
IGFBP5	0.08	Inhibit[32]	SDC1*	0.41	Promote[51]
EFEMP1	0.19	Promote[33]	COL1A1*	0.41	Promote[52]
ID1	0.23	Promote[34]	CDK1*	0.41	Promote[53]
VCAM1	0.27	Promote[35]	EPHA2	0.41	Promote[54]
LAMA4*	0.29	Promote[36]	WNT5B*	0.41	Promote[55]
CD24	0.30	Promote[37]	FBXO5	0.41	Inhibit[56]
IGKV1OR-2	0.30	/	FAM83D	0.42	Promote[57]
CCN2	0.30	Promote[38]	WWC1	0.43	Inhibit[58]
ANGPT1	0.31	Promote[39]	ANLN	0.44	Promote[59]
WDR63	0.31	Inhibit[40]	NR2F1	0.45	Promote[60]
ASTN2	0.31	/	MYLK	0.46	Promote[61]
CORO1A	0.33	Promote[41]	ACTL8	0.46	Promote[62]
TMSB4X	0.34	Promote[42]	GTSE1	0.46	Promote[63]
FGF7*	0.35	Promote[43]	PTN	0.46	Promote[64]
DCC*	0.36	/	ARHGEF39	0.47	Promote[65]
MATN2	0.37	Promote[44]	SRPX2	0.48	Promote[66]
FRMD5	0.39	Inhibit[45]	PRAG1	0.48	/
AMOTL2	0.39	/	NTNG1	0.48	/
ATOH8	0.39	Inhibit[46]	FOXG1	0.48	Promote[67]
PPARGC1A	0.39	/	GPNMB	0.48	Promote[68]
SCG2	0.39	/	NR4A2	0.48	Promote[69]
COL1A2*	0.39	Promote[47]	PRR5L	0.48	/
ACTA2	0.40	Promote[48]	ADA	0.49	Inhibit[70]
KANK1	0.40	Inhibit[49]	FERMT1	0.50	Promote[71]
PLK2	0.41	Inhibit[50]	TMEFF2	0.50	Inhibit[72]

* is the genes in the intersection of the two gene sets described as result section 3.5. The references in this table are shown in the end of this supporting information.

References in Supplementary Table 1 and 2

[1] Rébé C, Ghiringhelli F. Interleukin-1β and Cancer. Cancers (Basel). 2020 Jul 4;12(7):1791.
[2] Panneerselvam J, Jin J, Shanker M, Lauderdale J, Bates J, Wang Q, et al. IL-24 inhibits lung cancer cell migration and invasion by disrupting the SDF-1/CXCR4 signaling axis. PLoS One. 2015 Mar 16;10(3):e0122439.
[3] Deng L, Feng D, Ling B. Cyclooxygenase-2 promotes ovarian cancer cell migration and cisplatin resistance via regulating epithelial mesenchymal transition. J Zhejiang Univ Sci B. 2020 Apr.;21(4):315-326.
[4] Liu W, Wang W, Wang X, Xu C, Zhang N, Di W. Cisplatin-stimulated macrophages promote ovarian cancer migration via the CCL20-CCR6 axis. Cancer Lett. 2020 Mar 1;472:59-69.
[5] Gu Q, Luo Y, Chen C, Jiang D, Huang Q, Wang X. GREM1 overexpression inhibits proliferation, migration and angiogenesis of osteosarcoma. Exp Cell Res. 2019 Nov 1;384(1):111619.
[6] Liu X, Xu D, Liu Z, Li Y, Zhang C, Gong Y, Jiang Y, Xing B. THBS1 facilitates colorectal liver metastasis through enhancing epithelial-mesenchymal transition. Clin Transl Oncol. 2020 Oct;22(10):1730-1740.
[7] Zhou D, Zhao X, Yu M, Xu Y, Fu C, Zheng K, et al. Anti-migration and anti-invasion effects of 2-hydroxy-6-tridecylbenzoic acid is associated with the enhancement of CYP1B1 expression through activating the AMPK signaling pathway in triple-negative breast cancer cells. Nat Prod Res. 2020 Aug 11;1-5.
[8] Awan B, Turkov D, Schumacher C, Jacobo A, McEnerney A, Ramsey A, et al. FGF2 Induces Migration of Human Bone Marrow Stromal Cells by Increasing Core Fucosylations on N-Glycans of Integrins. Stem Cell Reports. 2018 Aug 14;11(2):325-333.
[9] Peng L, He K, Cao Z, Bi L, Yu D, Wang Q, Wang J, et al. CARD10 promotes the progression of renal cell carcinoma by regulating the NF-κB signaling pathway. Mol Med Rep. 2020 Jan;21(1):329-337.
[10] Luo X, Yao J, Nie P, Yang Z, Feng H, Chen P, et al. FOXM1 promotes invasion and migration of colorectal cancer cells partially dependent on HSPA5 transactivation. Oncotarget. 2016 May 3;7(18):26480-95.
[11] Polewski M D, Reveron-Thornton R F, Cherryholmes G A, Marinov G K, Aboody K S. SLC7A11 Overexpression in Glioblastoma Is Associated with Increased Cancer Stem Cell-Like Properties. Stem Cells Dev. 2017 Sep 1;26(17):1236-1246.
[12] Li W, Zhou D, Sun L, Xiao L, Liu Z, Zhou M, et al. LncRNA WTAPP1 Promotes Migration and Angiogenesis of Endothelial Progenitor Cells via MMP1 Through MicroRNA 3120 and Akt/PI3K/Autophagy Pathways. Stem Cells
. 2018 Dec;36(12):1863-1874.
[13] Kim Y, Lee S, Shim S, Kim A, Park J, Jang W, et al. Hyaluronic acid synthase 2 promotes malignant phenotypes of colorectal cancer cells through transforming growth factor beta signaling. Cancer Sci. 2019 Jul;110(7):2226-2236.
[14] Xu X, Yan Q, Wang Y, Dong X. NTN4 is associated with breast cancer metastasis via regulation of EMT-related biomarkers. Oncol Rep. 2017 Jan;37(1):449-457.
[15] Ando T, Kage H, Matsumoto Y, Zokumasu K, Yotsumoto T, Maemura K, et al. Integrin α11 in non-small cell lung cancer is associated with tumor progression and postoperative recurrence. Cancer Sci. 2020 Jan;111(1):200-208.
[16] Malric L, Monferran S, Delmas C, Arnauduc F, Dahan P, Boyrie S, et al. Inhibiting Integrin β8 to Differentiate and Radiosensitize Glioblastoma-Initiating Cells. Mol Cancer Res. 2019 Feb;17(2):384-397.
[17] Liang J, Sun Z. Overexpression of membranal SLC3A2 regulates the proliferation of oral squamous cancer cells and affects the prognosis of oral cancer patients. J Oral Pathol Med. 2021 Apr;50(4):371-377.
[18] Song X, Xie D, Xia X, Tan F, Pei Q, Li Y, et al. Role of SSH1 in colorectal cancer prognosis and tumor progression. J Gastroenterol Hepatol. 2020 Jul;35(7):1180-1188.
[19] Prasad C P, Chaurasiya S K, Guilmain W, Andersson T. 2WNT5A signaling impairs breast cancer cell migration and invasion via mechanisms independent of the epithelial-mesenchymal transition. J Exp Clin Cancer Res
. 2016 Sep 13;35(1):144.
[20] Xu Y, Liu H, Liu Z, Pan C, Yang X, et al. Sprouty2 suppresses progression and correlates to favourable prognosis of intrahepatic cholangiocarcinoma via antagonizing FGFR2 signalling. J Cell Mol Med. 2018 Nov;22(11):5596-5606.

[21] Sahu S N, Nunez S, Bai G, Gupta A, et al. Interaction of Pyk2 and PTP-PEST with leupaxin in prostate cancer cells. Am J Physiol Cell Physiol. 2007 Jun;292(6):C2288-96.
[22] Lin J, Tsai J, Chao T, Ma H, Liu W. Musashi-1 Enhances Glioblastoma Migration by Promoting ICAM1 Translation. Neoplasia. 2019 May;21(5):459-468.
[23] Fan X, Wang Y, Jiang T, Cai W, Jin Y, Niu Y, et al. B-Myb Mediates Proliferation and Migration of Non-Small-Cell Lung Cancer via Suppressing IGFBP3. Int J Mol Sci. 2018 May 16;19(5):1479.
[24] Baraya Y, Wong K K, Yaacob N S. Strobilanthes crispus inhibits migration, invasion and metastasis in breast cancer. J Ethnopharmacol. 2019 Apr 6;233:13-21.
[25] He T, Xiao Z, Xing Y, Yang H, Qiu H, Chen J. Tumor Suppressor miR-184 Enhances Chemosensitivity by Directly Inhibiting SLC7A5 in Retinoblastoma. Front Oncol. 2019 Nov 15;9:1163.
[26] Li P, Zhao S, Hu Y. SFRP2 modulates non-small cell lung cancer A549 cell apoptosis and metastasis by regulating mitochondrial fission via Wnt pathways. Mol Med Rep. 2019 Aug;20(2):1925-1932.
[27] Guo P, Moses-Gardner A, Huang J, Smith E R, Moses M A. ITGA2 as a potential nanotherapeutic target for glioblastoma. Sci Rep. 2019 Apr 17;9(1):6195.
[28] Lee H, Goodarzi H, Tavazoie S F, Alarcón C R. TMEM2 Is a SOX4-Regulated Gene That Mediates Metastatic Migration and Invasion in Breast Cancer. Cancer Res. 2016 Sep 1;76(17):4994-5005.
[29] Yan T, Lin Z, Jiang J, Lu S, Chen M, Que H. MMP14 regulates cell migration and invasion through epithelial-mesenchymal transition in nasopharyngeal carcinoma. Am J Transl Res. 2015 May 15;7(5):950-8.
[30] Zhang P, Shao G, Lin X, Liu Y, Yang Z. MiR-338-3p inhibits the growth and invasion of non-small cell lung cancer cells by targeting IRS2. Am J Cancer Res. 2017 Jan 1;7(1):53-63.
[31] Wu Q, Zhong H, Jiao L, Wen Y, Zhou Yi, Zhou J. MiR-124-3p inhibits the migration and invasion of Gastric cancer by targeting ITGB3. Pathol Res Pract. 2020 Jan;216(1):152762.
[32] Sureshbabu A, Okajima H, Yamanaka D, Tonner E, Shastri S, Maycock J, et al. IGFBP5 induces cell adhesion, increases cell survival and inhibits cell migration in MCF-7 human breast cancer cells. J Cell Sci. 2012 Apr 1;125(Pt 7):1693-705.
[33] Yin X, Fang S, Wang M, Wang Q, Fang R, Chen J. EFEMP1 promotes ovarian cancer cell growth, invasion and metastasis via activated the AKT pathway. Oncotarget. 2016 Jul 26;7(30):47938-47953.
[34] Hu X, Chen M, Li Y, Wang Y, Wen S, Jun F. Overexpression of ID1 promotes tumor progression in penile squamous cell carcinoma. Oncol Rep. 2019 Feb;41(2):1091-1100.
[35] Zhang D, Bi J, Liang Q, Wang S, Zhang L, Han F,et al. VCAM1 Promotes Tumor Cell Invasion and Metastasis by Inducing EMT and Transendothelial Migration in Colorectal Cancer. Front Oncol. 2020 Jul 23;10:1066.
[36] Shan N, Zhang X, Xiao X, Zhang H, Tong C, Luo X. Laminin α4 (LAMA4) expression promotes trophoblast cell invasion, migration, and angiogenesis, and is lowered in preeclamptic placentas. Placenta. 2015 Aug;36(8):809-20.
[37] Barash U, Spyrou A, Liu P, Vlodavsky E, Zhu C, Luo J, et al. Heparanase promotes glioma progression via enhancing CD24 expression. Int J Cancer. 2019 Sep 15;145(6):1596-1608.
[38] Wu Y, Li H, Zhao X, Jiao J, Tang D, Yan L, et al. Mesenchymal stem cell-derived CCN2 promotes the proliferation, migration and invasion of human tongue squamous cell carcinoma cells. Cancer Sci. 2017 May;108(5):897-909.
[39] Flores-Pérez A, Marchat L A, Rodríguez-Cuevas S, Bautista-Piña V, Hidalgo-Miranda A, Ocampo E A, et al. Dual targeting of ANGPT1 and TGFBR2 genes by miR-204 controls angiogenesis in breast cancer. Sci Rep . 2016 Oct 5;6:34504.
[40] Zhao K, Wang D, Zhao X, Wang C, Gao Y, Liu K, et al. WDR63 inhibits Arp2/3-dependent actin polymerization and mediates the function of p53 in suppressing metastasis. EMBO Rep. 2020 Apr 3;21(4):e49269.
[41] Qiao X, Niu X, Shi J, Chen L, Wang X, Liu J, et al. Wnt5a regulates Ameloblastoma Cell Migration by modulating Mitochondrial and Cytoskeletal Dynamics. J Cancer. 2020 Jul 11;11(18):5490-5502.
[42] Makowiecka A, Malek N, Mazurkiewicz E, Mrówczyńska E, Nowak D, Mazur A J. Thymosin β4 Regulates Focal Adhesion Formation in Human Melanoma Cells and Affects Their Migration and Invasion. Front Cell Dev Biol. 2019 Dec 23;7:304.

[43] Huang T, Wang L, Liu D, Li P, Xiong H, Zhuang L, et al. FGF7/FGFR2 signal promotes invasion and migration in human gastric cancer through upregulation of thrombospondin-1. Int J Oncol. 2017 May;50(5):1501-1512.
[44] Jiang H, Guo W, Yuan S, Song L. Matrilin-2 is a novel prognostic marker in osteosarcoma. Int J Clin Exp Pathol
. 2019 Oct 1;12(10):3752-3760.
[45] Wang T, Pei X, Zhan J, Hu J, Yu Y, Zhang H. FERM-containing protein FRMD5 is a p120-catenin interacting protein that regulates tumor progression. FEBS Lett. 2012 Sep 21;586(19):3044-50.
[46] Chen L, Yang J, Wang Y, Wu N, Li X, Li J, et al. ATOH8 overexpression inhibits the tumor progression and monocyte chemotaxis in hepatocellular carcinoma. Int J Clin Exp Pathol. 2020 Oct 1;13(10):2534-2543.
[47] Ao R, Guan L, Wang Y, Wang J. Silencing of COL1A2, COL6A3, and THBS2 inhibits gastric cancer cell proliferation, migration, and invasion while promoting apoptosis through the PI3k-Akt signaling pathway. J Cell Biochem. 2018 Jun;119(6):4420-4434.
[48] Lee H W, Park Y M, Lee S J, Cho H J, Kim D H, Lee J, et al. Alpha-smooth muscle actin (ACTA2) is required for metastatic potential of human lung adenocarcinoma. Clin Cancer Res. 2013 Nov 1;19(21):5879-89.
[49] Pu J, Shen J, Zhong Z, Yanling M, Gao J. KANK1 regulates paclitaxel resistance in lung adenocarcinoma A549 cells. Artif Cells Nanomed Biotechnol. 2020 Dec;48(1):639-647.
[50] Alafate W, Xu D, Wu W, Xiang J, Ma X, Xie W, et al. Loss of PLK2 induces acquired resistance to temozolomide in GBM via activation of notch signaling. J Exp Clin Cancer Res. 2020 Nov 11;39(1):239.
[51] Chen X, Zhao H, Chen C, Li J, He J, Fu X, et al. The HPA/SDC1 axis promotes invasion and metastasis of pancreatic cancer cells by activating EMT via FGF2 upregulation. Oncol Lett. 2020 Jan;19(1):211-220.
[52] Zhang Z, Wang Y, Zhang J, Zhong J, Yang R. COL1A1 promotes metastasis in colorectal cancer by regulating the WNT/PCP pathway. Mol Med Rep. 2018 Apr;17(4):5037-5042.
[53] Zhao S, Wang B, Ma Y, Kuang J, Liang J, Yuan Y. NUCKS1 Promotes Proliferation, Invasion and Migration of Non-Small Cell Lung Cancer by Upregulating CDK1 Expression. Cancer Manag Res. 2020 Dec 24;12:13311-13323.
[54] Garcia-Monclús S, López-Alemany R, Almacellas-Rabaiget O, Herrero-Martín D, Huertas-Martinez J, Lagares-Tena L, et al. EphA2 receptor is a key player in the metastatic onset of Ewing sarcoma. Int J Cancer. 2018 Sep 1;143(5):1188-1201.
[55] Zhang Y, Lin L, Jin Y, Lin Y, Cao Y, Zheng C. Overexpression of WNT5B promotes COLO 205 cell migration and invasion through the JNK signaling pathway. Oncol Rep. 2016 Jul;36(1):23-30.
[56] Zhang Z, Zhang G, Gao Z, Li S, Li Z, Bi J, et al. Comprehensive analysis of differentially expressed genes associated with PLK1 in bladder cancer. BMC Cancer. 2017 Dec 16;17(1):861.
[57] Mu Y, Zou H, Chen B, Fan Y, Luo S. FAM83D knockdown regulates proliferation, migration and invasion of colorectal cancer through inhibiting FBXW7/Notch-1 signalling pathway. Biomed Pharmacother. 2017 Jun;90:548-554.
[58] Chai Y, Li Q, Zhao H, Zhang Z, Yu X, Pang L, et al. SOX2 antagonizes WWC1 to drive YAP1 activation in esophageal squamous cell carcinoma. Cancer Med. 2019 Nov;8(16):7055-7064.
[59] Xu J, Zheng H, Yuan S, Zhou B, Zhao W, Pan Y, et al. Overexpression of ANLN in lung adenocarcinoma is associated with metastasis. Thorac Cancer. 2019 Aug;10(8):1702-1709.
[60] Gao X, Zheng M, Wang H, Dai L, Yu X, Yang X, et al. NR2F1 contributes to cancer cell dormancy, invasion and metastasis of salivary adenoid cystic carcinoma by activating CXCL12/CXCR4 pathway. BMC Cancer. 2019 Jul 29;19(1):743.
[61] Xia N, Cui J, Zhu M, Xing R, Lu Y. Androgen receptor variant 12 promotes migration and invasion by regulating MYLK in gastric cancer. J Pathol. 2019 Jul;248(3):304-315.
[62] Ma S, Wang X, Zhang Z, Liu D. Actin-like protein 8 promotes cell proliferation, colony-formation, proangiogenesis, migration and invasion in lung adenocarcinoma cells. Thorac Cancer. 2020 Mar;11(3):526-536.
[63] Wu X, Wang H, Lian Y, Chen L, Gu L, Wang J, et al. GTSE1 promotes cell migration and invasion by regulating EMT in hepatocellular carcinoma and is associated with poor prognosis. Sci Rep. 2017 Jul 11;7(1):5129.
[64] Elahouel R, Blanc C, Carpentier G, Frechault S, Cascone I, Destouches D, et al. Pleiotrophin exerts its

migration and invasion effect through the neuropilin-1 pathway. Neoplasia. 2015 Aug;17(8):613-24.
[65] Wang H, Li M, Tao X, Qian Y, Chen L, Tao G. ARHGEF39 promotes gastric cancer cell proliferation and migration via Akt signaling pathway. Mol Cell Biochem. 2018 Mar;440(1-2):33-42.
[66] Lin X, Chang W, Wang Y, Tian M, Yu Z. SRPX2, an independent prognostic marker, promotes cell migration and invasion in hepatocellular carcinoma. Biomed Pharmacother. 2017 Sep;93:398-405.
[67] Wu H, Qian C, Liu C, Xiang J, Ye D, Zhang Z. Role and mechanism of FOXG1 in invasion and metastasis of colorectal cancer. Sheng Wu Gong Cheng Xue Bao. 2018 May 25;34(5):752-760.
[68] Ren F, Zhao Q, Liu B, Sun X, Tang Y, Huang H, et al. Transcriptome analysis reveals GPNMB as a potential therapeutic target for gastric cancer. J Cell Physiol. 2020 Mar;235(3):2738-2752.
[69] Karki K, Li X, Jin U, Mohankumar K, Zarei M, Michelhaugh S K, et al. Nuclear receptor 4A2 (NR4A2) is a druggable target for glioblastomas. J Neurooncol. 2020 Jan;146(1):25-39.
[70] Niechi I, Uribe-Ojeda A, Erices J I, Torres Á, Daniel Uribe 5, José Dellis Rocha, et al. Adenosine Depletion as A New Strategy to Decrease Glioblastoma Stem-Like Cells Aggressiveness. Cells. 2019 Oct 30;8(11):1353.
[71] Liu C, Cai D, Sun F, Wu Z, Yue B, Zhao S, et al. FERMT1 mediates epithelial-mesenchymal transition to promote colon cancer metastasis via modulation of β-catenin transcriptional activity. Oncogene. 2017 Mar 30;36(13):1779-1792.
[72] Han H, Zhan Z, Xu J, Song Z. TMEFF2 inhibits pancreatic cancer cells proliferation, migration, and invasion by suppressing phosphorylation of the MAPK signaling pathway. Onco Targets Ther. 2019 Dec 23;12:11371-11382.
